# Time Course and Mechanisms Underlying Standing Balance Recovery Early After Stroke: Design of a Prospective Cohort Study With Repeated Measurements

**DOI:** 10.3389/fneur.2022.781416

**Published:** 2022-02-21

**Authors:** Jonas Schröder, Wim Saeys, Laetitia Yperzeele, Gert Kwakkel, Steven Truijen

**Affiliations:** ^1^Research Group MOVANT, Department of Rehabilitation Sciences and Physiotherapy, Faculty of Health Sciences, University of Antwerp, Wilrijk, Belgium; ^2^M^2^OCEAN Lab, The Multidisciplinary Motor Centre Antwerp, Faculty of Health Sciences, University of Antwerp, Edegem, Belgium; ^3^RevArte Rehabilitation Hospital, Edegem, Belgium; ^4^Department of Neurology, Neurovascular Reference Center, Antwerp University Hospital, Edegem, Belgium; ^5^Research Group Translational Neurosciences, Faculty of Medicine and Health Sciences, University of Antwerp, Wilrijk, Belgium; ^6^Department of Rehabilitation Medicine and Amsterdam Neuroscience, Amsterdam Movement Sciences, Amsterdam University Medical Centre, Amsterdam, Netherlands; ^7^Department of Physical Therapy and Human Movement Sciences, Feinberg School of Medicine, Northwestern University, Chicago, IL, United States; ^8^Department of Neurorehabilitation, Amsterdam Rehabilitation Research Centre, Reade, Amsterdam, Netherlands

**Keywords:** stroke recovery, motor recovery, force plate analysis, longitudinal regression analysis, standing balance, prospective longitudinal cohort study

## Abstract

**Introduction:**

Although most stroke survivors show some spontaneous neurological recovery from motor impairments of the most-affected leg, the contribution of this leg to standing balance control remains often poor. Consequently, it is unclear how spontaneous processes of neurological recovery contributes to early improvements in standing balance.

**Objective:**

We aim to investigate (1) the time course of recovery of quiet stance balance control in the first 12 weeks poststroke and (2) how clinically observed improvements of lower limb motor impairments longitudinally relate to this limb's relative contribution to balance control.

**Methods and Analysis:**

In this prospective longitudinal study, a cohort of 60 adults will be recruited within the first 3 weeks after a first-ever hemispheric stroke and mild-to-severe motor impairments. Individual recovery trajectories will be investigated by means of repeated measurements scheduled at 3, 5, 8, and 12 weeks poststroke. The Fugl-Meyer Motor Assessment and Motricity Index of the lower limb serve as clinical measures of motor impairments at the hemiplegic side. As soon as subjects are able to stand independently, bilateral posturography during quietly standing will be measured. First, the obtained center-of-pressure (COP) trajectories at each foot will be used for synchronization and contribution measures that establish (a-)symmetries between lower limbs. Second, the COP underneath both feet combined will be used to estimate overall stability. Random coefficient analyses will be used to model time-dependent changes in these measures and, subsequently, a hybrid model will be used to investigate longitudinal associations with improved motor impairments.

**Discussion:**

The current study aims to investigate how stroke survivors “re-learn” to maintain standing balance as an integral part of daily life activities. The knowledge gained through this study may contribute to recommending treatment strategies for early stroke rehabilitation targeting behavioral restitution of the most-affected leg or learning to compensate with the less-affected leg.

## Introduction

### Background

Stroke is a major cause for acquired serious disability in adults worldwide (1). It is well known that stroke affects standing balance, for example posturographic studies revealed that stroke survivors show increased body sway reflected by center-of-pressure (COP) movements (2–5). Recovering the ability to maintain standing balance is seen as a prerequisite for regaining walking independence (6), and residual deficits are associated with limited community ambulation (7, 8) and falls (9–11). Accordingly, falls remain a major problem with approximately 50 to 70% of community-dwelling stroke survivors experiencing a fall recently after being discharged home from rehabilitation facilities (9, 10, 12). As such, deficient standing balance control may contribute to chronic disability and fall-related injury resulting in greater economic costs due to the need of care (13) and poor quality of life (14).

Improving stroke rehabilitation services requires a profound understanding about the behavioral changes underlying impaired as well as improved balance control execution, together with the time windows in which they develop. This may enable clinicians to define appropriate treatment targets and rehabilitation goals early onwards. From this perspective, particularly explorative longitudinal research with repeated measurements early in time is warranted to distinguish between behavioral restitution and compensation when control of standing balance is restored (15, 16). As stroke recovery is a complex process that likely involves both spontaneous and learning-dependent mechanisms (17, 18), this research should be based on a commonly shared framework for classifying and using uniform terminology.

The WHO's international classification of function, disability and health (ICF) model may serve as such framework by categorizing the consequences of stroke in terms of body functions, activities and participation (17, 19). Following the ICF, we will discuss available literature and, subsequently, the design of an ongoing observational study into the time course and mechanisms underlying standing balance recovery. For this purpose, we distinguish between recovery of motor impairments of the most-affected lower limb (i.e., ICF level of body functions) and an improved ability to maintain standing balance as an integral part of daily life activities (i.e., ICF level of activities).

#### Time Course of Lower Limb Recovery

Unfortunately, only few longitudinal studies have been designed to investigate prospectively the time course of recovery of lower limb function (20–23) and activities (24–26) early after stroke. These studies suggest that recovery of motor impairments, such as synergy-dependency (20, 22, 23) and weakness (21, 23), mainly occurs in the first 5 weeks and levels off between 8 to 12 weeks poststroke (20–22). However, most survivors experience residual impairments in dissociating voluntary foot and leg movements from intra-limb synergies (27, 28) and muscle strength (23) which is associated with a decreased contribution of this leg to standing balance control (29–32). These early time-dependent changes remain poorly understood, but can be conceptualized as spontaneous neurological recovery as reflected by the passage of time (23).

Similar regularities in recovery patterns of activities involving the lower limbs have been shown. Likewise, most improvements in walking (6, 24–26) and other daily life activities (21, 23) were seen in the first weeks and regaining standing balance is found imperative to such recovery (6). One might suggest that spontaneous neurological recovery is associated with, if not determinant for these rapid improvements. This would mean that the ability to complete these tasks is restored with the same movement repertoire, or quality, the patient had before the stroke (i.e., behavioral restitution) (18, 19, 33). However, previous longitudinal research is largely limited to outcomes showing the mere accomplishment on these tasks. As these measures are unable to discriminate *how* the task is performed (19, 34, 35), little has been learned so far about the time course of recovery regarding qualitative aspects of movement when standing balance is restored.

#### Mechanisms Underlying Recovery of Standing Balance Control

As recently suggested (28), a number of severely affected subjects fail to show neurological recovery (28), leaving them entirely dependent on learning to use alternative ways to maintain their standing balance (i.e., behavioral compensation). However, even “well-recovered” patients with near normal clinical scores may show a disproportional reliance on the less-affected leg to balance control (31, 36). This is illustrated by more recent posturographic studies showing that bilateral COP displacements at the feet separately, as a reflection of the anticipatory modulation of ankle muscle activity aiming to minimize body sway (37, 38), are poorly synchronized (32, 39) and unequal (29–31, 40, 41) as compared to healthy controls. It seems that neurological recovery, even if occurring, is rarely complete and an even larger portion of survivors may depend on using compensation strategies to deal with residual impairments.

In favor of this notion, few longitudinal studies did show that standing balance improves in many patients *without* any concomitant improvements in anticipatory control of most-affected leg muscles in response to rapid arm movements (34, 42) or *without* restitution of symmetric exertion of corrective COP movements during unperturbed, quiet stance (2, 30, 40). However, these studies did unfortunately not start in the first weeks and assessed subjects at only few arbitrarily chosen time-points, leaving longitudinal associations with neurological recovery entirely underexplored.

As it remains unknown how stroke survivors “re-learn” to maintain standing balance in the early recovery phase when motor functions at the hemiplegic side improve, there is a need for early-started longitudinal research incorporating sensitive measures of task performance next to clinical scales. These studies may eventually progress our understanding of how spontaneous neurological recovery contributes to the re-acquisition of bipedal balance skills early after stroke onset.

### Objectives

In the current manuscript, we describe an observational cohort study that aims to prospectively investigate the time course of recovery of quiet stance balance control in the first 12 weeks after a first-ever hemispheric stroke. The relatively low functional demands of this condition (as compared to perturbed stance or walking) enables us to describe the process of balance skill re-acquisition even in more-severely impaired subjects and in the time window of spontaneous neurological recovery. Simultaneously, we will clinically observe the time course of recovery of lower limb motor impairments to investigate longitudinal associations with this leg's contribution to balance control.

The pre-defined research questions and hypotheses are:

- What is the time course of recovery in the first 12 weeks poststroke in terms of balance control (a-)symmetries and overall stability during quiet stance? (i.e., project A).

First, we hypothesize that improvements in balance control symmetries toward values seen in healthy controls, as assessed through bilateral posturography (i.e., between-limb synchronization and contribution measures of corrective COP movements), are restricted to the first 5 weeks poststroke. Second, we hypothesize that an improved ability to maintain a stable standing position following traditional posturographic measures of body sway (i.e., stance stability) may develop beyond the time window and up until 12 weeks poststroke. As such, these later changes are hypothesized to reflect compensatory body stabilization exerted through the less-affected leg.

- How are improvements in muscle synergies and ankle strength of the most-affected leg longitudinally associated with this leg's contribution to quiet stance balance control in the first 12 weeks poststroke? (i.e., project B).

First, we hypothesize that clinical improvements in muscle synergies (i.e., Fugl-Meyer Motor Assessment) and strength (i.e., Motricity Index) at the hemiplegic side are mainly seen within the first 5 weeks poststroke, in accordance with previous longitudinal studies investigating time-dependent change (20, 23). Second, we hypothesize to find within this time window longitudinal associations between recovery on these scales and improved between-limb symmetry in balance control. This means that clinically observed improvements in motor impairments relate over the first weeks to an improved ability of this leg to contribute to corrective COP movements while standing quietly.

## Methods

The protocols of prospective repeated-measurement studies from which participants will be recruited for this longitudinal investigation are registered online (ClinicalTrials.gov Identifier: NCT03728036; NCT03727919). These studies are designed and executed in adherence to the STROBE statements and were approved by the local Ethics Committee of the Antwerp University Hospital (Edegem, Belgium) in accordance with the declaration of Helsinki (main ethics committee protocol number: 18/25/305; Belgium trial registration number: B300201837010). The responsible ethics committees of all other involved clinical sites were asked for advice and additional approval before the study started.

### Participants

In total, this study will include a cohort of 60 patients after a first-ever hemispheric stroke of ischemic or hemorrhagic cause within, at most, 3 years of recruitment. This cohort will be recruited from the stroke units at the University Hospital Antwerp (Edegem, Antwerp, Belgium), the GZA Hospital campus St Augustinus (Wilrijk, Antwerp, Belgium) and the General Hospital Geel (Antwerp, Belgium). All cooperating hospitals are located in the larger Antwerp region in Flanders, Belgium. Stroke survivors will be screened and asked to participate as soon as possible and within the first 3 weeks after stroke onset. Only survivors who are, or will be admitted to one of the involved rehabilitation facilities (RevArte, Edegem, Antwerp, Belgium or the rehabilitation hospital Geel, Geel, Antwerp, Belgium) for inpatient treatment (eventually followed by outpatient treatment) are considered for inclusion, as the repeated measurements will be performed there. In addition, ten middle-aged (i.e., 45 to 65 years) healthy adults (gender equally distributed) with no known musculoskeletal or neurological injury or illness that may affect balance control will be recruited to serve as controls for the interpretation of posturographic measures of quiet stance balance control.

Before entry, an information brochure about the study's aim, content and potential risks, together with information about the investigators is provided to each prospective subject (and if adequate to her/his family). If the subject feels sufficiently informed and agrees to participate, an informed consent is signed and eligibility is determined according to the following criteria.

#### Inclusion Criteria

- A first-ever, CT- or MRI-confirmed, supra-tentorial stroke in the anterior or middle cerebral arteria territory of ischemic or hemorrhagic cause;
- Impaired motor functions of the lower limb, defined as a score of > 0 on the NIHSS item 6 (i.e., at least “drift of the leg within 5 s without a fall”) within 3 days after stroke onset (or self-reported if data is missing), and a MI-LE score of ≤ 91 (i.e., at least “movement against resistance but weaker” in one item) at the moment of inclusion;
- Age between 18 and 90 years;
- Sufficient motivation to participate.

#### Exclusion Criteria

- Dependent in daily life activities before stroke onset, defined as a pre-morbid Functional Ambulation Category score of <4 and a modified Rankin Scale score of >1;
- Having a preexisting orthopedic or neurological condition that affects motor functions of the lower limbs and/or standing balance control;
- Severe cognitive or communication deficits that may hinder informed consent and execution of the study;
- Not Dutch, German or English speaking prior to the stroke incident.

### Design

The current recovery study is a non-interventional observational study meaning that no systematic interventions are applied next to usual care. For the duration of inpatient rehabilitation, this consists of 30- to 60-min sessions of physical and occupational therapy each workday following local guidelines.

After eligible subjects are included within the first 3 weeks poststroke, serial measurements will be scheduled at 3, 5, 8, and 12 weeks poststroke. Clinical evaluations are performed by the same assessor for each participant to decrease the effect of inter-rater variability when measuring within-subject change. In total, two physical therapists are available for clinical testing who underwent a training period to standardize assessment procedures prior to the study start. As soon as subjects are able to stand independently barefoot over at least 30 seconds, bilateral posturography will be performed during a standardized quiet stance task. For this purpose, either two separate force-plates (Type OR 6-7, AMTI, MA, US; measurement frequency 1 kHz) arranged in a side-by-side configuration as part of a stationary movement analysis laboratory (*M*^2^*OCEAN*, University Hospital Antwerp, Edegem, Belgium) or a portable pressure-plate system (0.5 m footscan 3D, RS Scan, Belgium; measurement frequency 500 Hz) will be used. This provides the advantage of executing this study in multiple (clinical) settings to facilitate recruitment and continuous data acquisition. Importantly, repeated within-subject measurements are always performed with the same equipment and these measurements are performed by a trained physiotherapist who is educated in using the measuring instruments.

### Data Collection and Processing

During the uptake procedure, the participant's demographics including sex and age together with information about the stroke lesion in terms of type (i.e., infarct or bleeding) and side (i.e., left or right hemisphere affected) is collected. Length of stay in the rehabilitation hospital and discharge destination will be recorded throughout the study.

Before each balance assessment, the subject's body weight and length is measured. Next, the subject's bare feet will be positioned in parallel with a 8.4 cm heel-to-heel distance and in a 9° toe-out angle on the measuring plate (40). Manual support will be provided during standing up until the subject feels comfortable to stand independently. Now, the subject is instructed to maintain this position for 40 s with the arms alongside the trunk and the eyes open. They are asked to look straight ahead at a visual target at an approximately 2-meter distance. Subjects are encouraged to adopt a spontaneous, stable posture rather than standing as symmetric as possible. Three trials will be performed and if the subject got distracted, lost balance or moved the arms or head in a way that is not related to balance, the trial is excluded.

From each eligible trial, the first 10 s will be removed to prevent the influence of starting effects. Previously it was shown that 30 s quiet stance registration yield excellent test-retest reliability (43). Limb-specific COP trajectories will be calculated based on raw force components and low-pass filtered using a zero-lag, second-order Butterworth filter with a 10 Hz cutoff frequency. For the calculation of synchronization and contribution measures, these trajectories will be split into an anteroposterior (AP) and mediolateral (ML) signal based on the orientation of the feet by rotating the reference system. The COP underneath both feet combined will be used to estimate overall stance stability. For this purpose, we focus on velocity parameters as these are shown to have greatest reliability (44, 45) which has been confirmed for bilateral posturography in stroke survivors (46). These studies also show that usually two to three trials are sufficient to reach reliable data (45, 46) which is of relevance for this project since instability is pronounced early after stroke allowing to acquire only few standing attempts. All data processing (and parameter calculation, as documented below) will be done by using custom-made Matlab scripts (The MathWorks Inc., MA, USA).

### Outcomes

**Figure 1:**
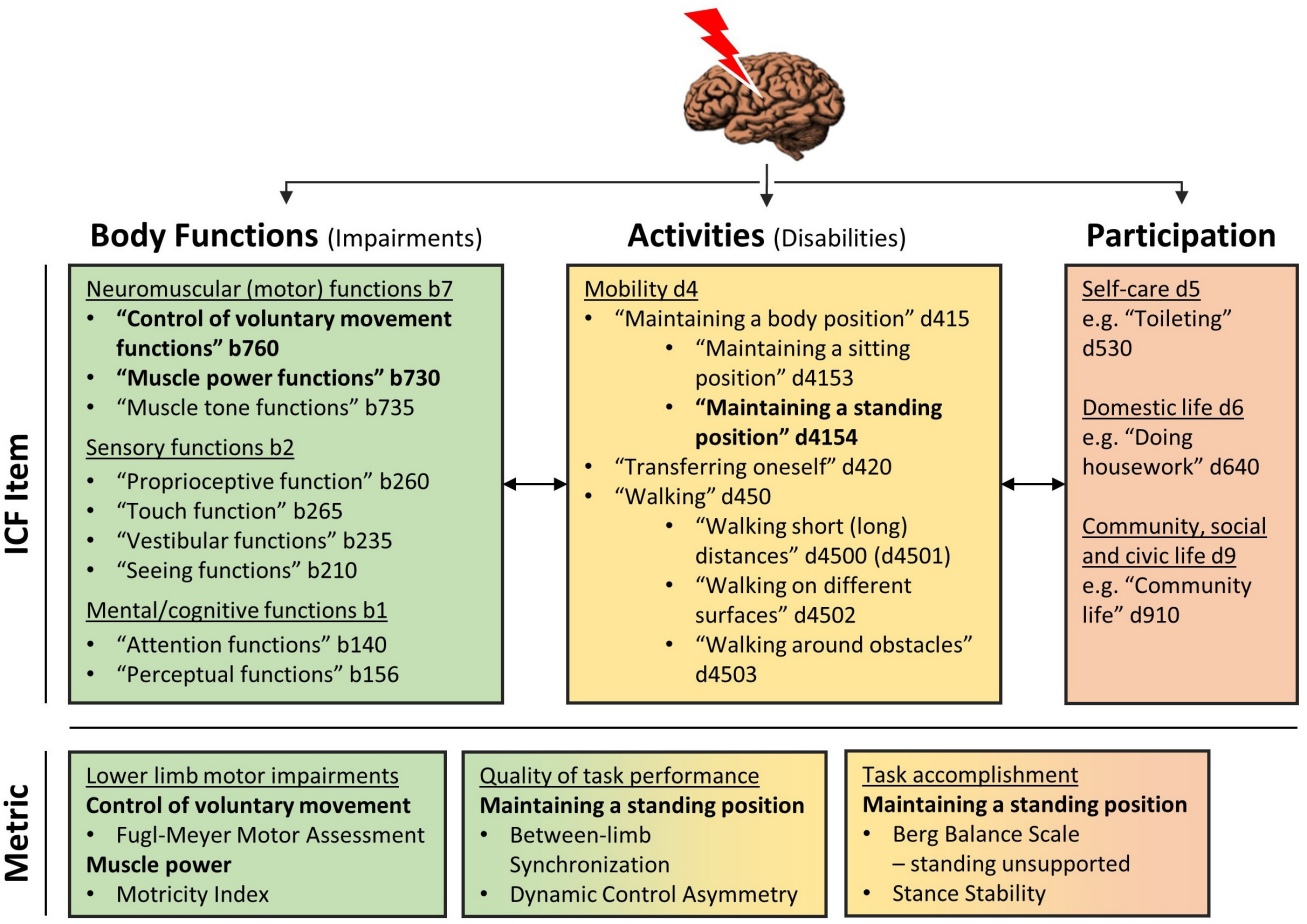
ICF model. The upper panel provides an overview of ICF items that are required for (i.e., level of body functions, colored green) and dependent on (i.e., level of activities, colored yellow, and participation, colored red) standing balance control. The items that are directly assessed in the current study are highlight in bolt. In the lower panel metrics corresponding to these items of interest are provided. The color-coding indicates that metrics of quality of task performance and task accomplishment are both primarily situated on the level of activities, whereas the first indicates how movement execution functions (i.e., level of body functions) are assembled to execute a balance task. Contrary, task accomplishment means if a subjects is successful in maintaining standing balance, irrespective of the underlying control strategy, as a determinant of independence in daily life activities and participation (i.e., level of participation).

Poststroke recovery is a complex matter and the inconsistent use of terminology in the literature creates opportunities for confusion. Therefore, we first define the chosen outcome variables following the ICF framework (see Figure 1) and differentiate between dependent and independent variables for subsequent analyses.

#### Independent Variables of Outcome

The main independent variables for modeling longitudinal associations with balance control measures (i.e., project B) are time-dependent, or dynamic, and situated on the ICF level of body functions (see Table 1). On this level, recovery means the restitution of rudimental functions of movement execution (19, 35), such as synergy-dependency and strength which we define as follows.

**Table 1:** Measurements per assessment occasion.

**Domain/measure**	**Measurement occasions (time poststroke)**				
	**<3 weeks (i.e., inclusion)**	**3 weeks**	**5 weeks**	**8 weeks**	**12 weeks**
**Uptake procedure**					
Informed consent	X				
**Independent variables: Potential covariates/confounders**					
Demographics: Gender (m/f/x), Age (years)	X				
Lesion characteristics: Type (infarct/bleeding), Side (left/right hemisphere)	X				
Anthropometrics: Body Height (mm), Body Weight (kg)^*^	X	X^*^	X^*^	X^*^	X^*^
Other: Equipment (force-plates/pressure-plate)	X				
**Independent variables: Body functions level-Lower limb motor impairments**					
Fugl Meyer assessment–lower extremity		X	X	X	X
Motricity Index–lower extremity		X	X	X	X
**Dependent variables: Activity level-Quality of task performance**					
Between-limb synchronization		X	X	X	X
Dynamic control asymmetry		X	X	X	X
**Dependent variables: Activity level-Task accomplishment**					
Berg Balance Scale–standing unsupported item		X	X	X	X
Stance stability		X	X	X	X

A scheme of the obtained metrics and items per assessment occasion is presented. Note, body weight (as marked with a ^*^) is the only potential additional covariate that is considered time-dependent and is therefore assessed at each follow-up occasion.

##### Dynamic Variables

Although different definitions and constructs of muscle synergies exists in the literature (47), we refer to synergies as the clinical phenomenon of “pathological intra-limb synergies” meaning the loss of independent joint control leading to the emergence of stereotypical flexor and extensor movements (48) (i.e., ICF item b760 “Control of voluntary movement functions”). As such, muscle synergies are defined as an “increased co-activation between muscles in the paretic limb that can be elicited voluntarily” (48). Second, muscle strength is defined as the ability to produce a “maximum voluntary force or torque” through a muscle or muscle group contraction around a single joint (i.e., ICF item b730 “Muscle power functions”) (49). The following standardized clinical scales will be used to address these functions.

- **Fugl Meyer motor assessment–lower extremity (FMA-LE)**: Muscle synergies will be assessed by means of scores on the FMA-LE, ranging from 0 (i.e., unable to move the limb or evoke tendon reflexes) to 34 points (i.e., able to selectively flex the knee and ankle joint in standing and normal reflexes). This scale has been reported to have excellent inter-rater [Pearson's *r* = 0.96 (50); ICC = 0.91, 0.97–1.0 95% CI (51)] and intra-rater reliability [ICC = 0.99, 0.91–1.0 95% CI (51)] when assessed in stroke survivors. Moreover, we use the standardization method of See et al. (52) to improve scoring consistency.
- **Motricity Index–lower extremity (MI-LE)**: Muscle strength in hip flexion, knee extension and ankle dorsiflexion direction will be assessed with the MI-LE. Active range of motion and force against manual resistance will be compared between both sides and evaluated with scores varying between 0 (i.e., no voluntary muscle contraction) and 33 points (i.e., full movement against gravity and equal strength) for each item. This scale has shown excellent intra-rater [ICC = 0.93, 0.84–0.97 95% CI (53); Spearman's rho = 0.87 (54)] and inter-rater reliability [Spearman's rho = 0.87 (54)] in stroke survivors.

##### Fixed Variables

Fixed, or time-dependent variables that are hypothesized to be potential confounders will be used as additional covariates. This includes the subject's age and gender at inclusion, as well as their Body Mass Index (BMI) (see Table 1). Body weight is serially assessed at each occasion and if significant fluctuations are seen over time, the BMI may be added as a dynamic variable. In addition, the stroke type and side will be added. Lastly, the used equipment for serial subject-specific measurements will be evaluated as a potential confounder.

#### Dependent Variables of Outcome

Dependent variables of outcome will be investigated in this study on the time course of recovery (i.e., project A) and longitudinal associations (i.e., project B). These outcomes are situated on the ICF level of activities where recovery comprises a general improvement in the ability to execute purposeful movements in a task context (19, 35). In the current study, the task of maintaining a quiet standing posture (i.e., ICF item d4154) will be evaluated which is defined as “the ability to control the body's center-of-mass relative to the base of support in fairly predictable and non-changing conditions” (55). For this purpose, we use and distinguish metrics addressing the quality of performance from those showing the mere accomplishment on this task.

##### Quality of Task Performance

Quality of task performance is “defined through a direct comparison of a patient's motor execution of a task […] to able-bodied control subjects” (35). This means that the closer the movement matches those seen in controls, the better the quality. With regard to standing balance, quality of performance is best reflected by measures that establish the (a-)symmetry between the most- and less-affected side considering that healthy balance control is characterized by equal output generated through the legs in the form of corrective COP movements (39, 56, 57). For this purpose, synchronization and contribution measures will be calculated that show how well both limbs act together (32, 39, 56) and are equally involved (31, 57) in maintaining standing balance. As COP in the ML direction is less meaningful for bipedal balance control (56), we focus on the sagittal plane.

- **Between-limb synchronization:** Between-limb synchronization is a measure of the temporal structure and similarity between bilateral COP (39, 56). For this purpose, the mean position is subtracted from left and right AP COP trajectories and, next, a cross-correlation at zero-phase leg on a frame-by-frame basis is calculated. This measure therefore shows how well COP displacements are alike, or synchronized.
- **Dynamic control asymmetry (DCA)**: DCA is a symmetry index of the root mean square (RMS) of the AP COP velocities for each leg separately (31, 57). A score of zero indicates equal contribution of both legs to balance control and positive or negative values indicating a relatively larger involvement of the less- or most-affected leg, respectively. We use the following equation: Symmetry index: 2x (RMS COP AP velocity *less-affected*- RMS COP AP velocity *most-affected*) / (RMS COP AP velocity *less-affected* + RMS COP AP velocity *most-affected*).

##### Task Accomplishment

Metrics on task accomplishment are designed to show if a patient re-acquired the ability to complete the task irrespective of the underlying control strategy. With regard to standing balance, this is exemplified first by the level of independence following clinical scales and second by (traditional) posturographic measures of body sway that show how well a subject can stabilize their center-of-mass within the base of support. Therefore, these outcome variables will be used to address the process of regaining and optimizing the ability to maintain standing balance.

- **Berg Balance Scale - standing unsupported item (BBS-s)**: The BBS-s assesses the ability to maintain assesses the ability to maintain a quiet standing posture without using the arms or support by another person. A score is assigned based on the level of independence needed to complete this task, where a score of 0 indicates no standing ability and a score varying between 1 and 4 indicate independent stance over 30 s to 2 min. The BBS has been reported to have excellent internal consistency as reported by a systematic review (58).
- **Stance stability**: To investigate overall stability when subjects attempt to stand quietly, we will calculate the root mean square (RMS) velocity of the net COP (i.e., combining two feet together without correction for feet orientation) in AP and ML direction. As the position of the net COP and the vertical representation of the body's center-of-mass correlate (59) these metrics reflect the ability to stand with minimal body sway.

### Data Analysis

#### Descriptive Analysis

Subject's demographics at baseline together with lesion characteristics will be descriptively analyzed. The length of stay in the inpatient rehabilitation facility and discharge destination will be reported. In addition, adherence to the study protocol will be illustrated by reporting the number of subjects leaving the study prematurely and reasons for dropping out entirely or missing assessments. We will use the BBS-s to show how soon subjects recovered standing balance and were able to participate in posturographic assessments.

#### Statistical Analysis

##### Project A

In project A, we aim to investigate time-dependent changes in metrics of quiet stance balance control. To estimate how each parameter is changing as a function of time poststroke, we use a random coefficient analysis (or mixed model analysis) with “time” of measurements as the main fixed effect (JMP Pro, version 15). Additionally, “time” will be entered as an independent covariate in form of an subject-specific slope (i.e., the interaction term “participant^*^time”) to adjust for dependency of repeated observations. However, the greatest advantage of this method is its flexibility in dealing with missing values. The latter may result from subjects being unable to stand at first occasions, being unavailable due to hospital discharge or transfers, or by no longer corresponding to eligibility criteria for example due to a recurrent stroke or other sudden medical condition (potentially) affecting outcome variables. Moreover, the value on the addressed metric at 3 weeks poststroke will be added to account for inter-subjects variability.

In addition, fixed covariates that are hypothesized to be potential confounders will be entered in the model. This includes “gender,” “age” and “BMI” considering their influence on standing balance control. [i.e., females and elderly tend to show greater body sway (60) and obesity is associated with instability (61)]. Second, stroke “side” and “type” will be added, as right-sided lesions typically result in greater balance deficits (62) and subjects with hemorrhagic strokes may display delayed recovery (63). Lastly, “equipment” is added as a potential confounder considering technical variations in measuring instruments.

Statistical analysis of the difference in each measure of quiet stance balance control between subjects and healthy controls will be performed using the Mann-Whitney U test for each measuring system separately.

##### Project B

After describing the recovery time course, we aim to investigate longitudinal associations between motor impairments in terms of leg muscle synergies (i.e., FMA-LE) and ankle strength (i.e., ankle item of the MI-LE) serving as independent variables, and the DCA which is the dependent variable. First, we analyze the pattern of neurological recovery following these clinical scales by using similar methods as outlined above. Second, we will apply a recently discussed hybrid model (64) to investigate longitudinal associations over the first 12 weeks poststroke. This method has the advantage of disentangling the between- and within-subject effects of this relation.

The between-subjects covariate score is determined as the individual average value over time of the independent variables which reveals the association irrespective of the development over time. On the other hand, the within-subject covariate is calculated as the observed value at each time point minus the individual average (i.e., deviation score). By this, we can estimate whether longitudinal changes of the dependent and independent variables within a subject are associated, i.e., are increasing scores on the FMA-LE or MI-LE ankle item related to changes following the DCA. Similar covariates will be entered in the model and tested on significance.

For all statistical tests, the likelihood ratio test will be used to examine the need to enter random effects into the model and the Wald test will be used to obtain *P*-values for regression coefficients in the final model. A 2-tailed significance level of 0.05 will be used for all analyses.

### Sample Size Justification

To the best of our knowledge, this is the first study to prospectively investigate changes in the variables over time early after stroke. This certainly limits the effectiveness of a sample size determination based on a power analysis. Therefore, we determine and justify our sample size of *n* = 60 based on recruitment (2.2 participants/month) and drop-out (15%) rates as seen during the first year of recruitment. Considering that we will include not more than 3 to 4 (main) covariates in our random coefficient and hybrid models, we meet the “rule of thumb” saying that 10 subjects per variable are sufficient to perform bivariate and multivariate regression analyses.

### Trial Status

Participant recruitment began in January 2019 in the University Hospital Antwerp and the RevArte rehabilitation hospital, in the GZA Sint-Augustinus hospital in April 2019 with a temporal suspension of recruitment in all involved sites between March and September 2020 due to Covid-19 measures. In response, an additional partnership with the General Hospital Geel in January 2021 was set-up. By now (October 2021), 52 stroke survivors were recruited indicating feasibility of reaching the desired sample size within the proposed recruitment period.

## Discussion

In this manuscript, we describe the design of an ongoing observational study with repeated measurements in time. This study aims to prospectively investigate individual recovery trajectories in a cohort of 60 mild-to-severely impaired subjects early after a first-ever, ischemic or hemorrhagic hemispheric stroke. Bilateral posturography will be used to measure balance control (a-) symmetries and overall stability during a quiet stance task to investigate the time course of recovery following these posturographic measures within subjects (i.e., project A) and, subsequently, longitudinal associations with recovery of lower limb motor impairments (i.e., project B). The knowledge gained through this study may contribute to our understanding of how progress of time as a reflection of spontaneous recovery contributes to regaining standing balance control through the most-affected leg, as well as dependency on compensatory stabilization exerted through the less-affected leg.

### Time Course of Lower Limb Recovery

Recent upper limb recovery studies (65, 66) attest to the effectiveness of incorporating sensitive and specific task performance measures into longitudinal research. Based on repeated kinematic measures of a reaching task, it was shown that recovery of movement quality with the hemiplegic arm plateaus in most patients over the first 5 weeks poststroke (65, 66). This suggests that further task improvements are most likely explained on the basis of compensatory mechanisms, such as increased trunk movements to assist arm and hand transport (19). If assuming that neurological recovery regarding the upper and lower limb develops in parallel, as suggested by previous clinical research (20, 22, 23), a similar distinct time window of behavioral restitution of standing balance control through the most-affected leg might be expected.

Previous posturographic studies already showed that the DCA shows little tendency to diminish over inpatient rehabilitation (40) resulting in a poor contribution of the most-affected leg to balance control (29, 31, 32, 41). Acknowledging that recovery of standing balance may extend far beyond the first weeks, e.g., improvements are seen in response to specific training even in the chronic stage (67), it seems that learning to compensate with the intact leg drives the reacquisition of functional balance skills after stroke. In favor of this notion, few longitudinal studies report consistent improvements over the first months in timed muscle activation with the less-affected leg to effectively correct balance after perturbations (34, 42, 68). Simultaneous changes at the hemiplegic side are often absent (34, 42, 68). However, how progress of time contributes to the relative involvement of the most-affected limb to balance control and, consequently, when such compensation need to emerge has hardly been investigated early after stroke. This makes project A of the current study unique.

### Mechanisms Underlying Recovery of Standing Balance Control

When standing, even smallest movements of the body must be corrected to avoid excessive sway and eventually a fall. As such, fine motor control is demanded for effective balance control. However, balance-related leg muscle activation is often disturbed after stroke. Abnormal intra-limb coordination patterns (68, 69) and delayed muscle onset (34, 42, 68) characterize reactive balance control through the hemiplegic leg, and inter-limb muscle activity about the ankle joints is poorly synchronized (11, 32, 39) and unequal exerted (2, 29–31, 40) during unperturbed stance. What determines poor muscle control as seen during balancing tasks is unknown, but may involve synergy-dependency (30, 31).

Already in 1951, Twitchell showed based on meticulous clinical observations that regaining control over the hemiplegic limb goes through synergy-dependent stages (48). This is later confirmed by longitudinal studies (20, 22, 23) showing progressively increasing scores on the FMA-LE over the first weeks poststroke. One might suspect a relationship between such clinical gains and an improved ability to execute functional movements with this leg, but this has been investigated cross-sectionally only with regard to standing balance (30–32). Although a relation was suggested, it is considered weak (31, 32). It was recently even shown that patients with near normal scores on the FMA-LE may show considerable control asymmetries (31). Although speculative, one may suggest that subtle fine motor control impairments go undetected by these scales, while it remains entirely unknown how this relation develops early after stroke. Since this knowledge has implications for rehabilitation practice, project B can be regarded as being innovative and of clinical relevance.

### Clinical and Scientific Significance

It is important for rehabilitation clinicians to distinguish improved standing balance resulting from behavioral restitution of the most-affected limb and compensatory stabilization through the less-affected limb. Historical treatment concepts strive to restore normal movement patterns (70), and even recent therapies such as feedback-based balance training involve teaching patients to stand as symmetric as possible (71). This might be questioned acknowledging that many stroke survivors seem unable to restore symmetric balance control strategies (31, 40). These patients may even benefit from some asymmetric loading to make corrective COP movements at the less-affected side more effective (57). From this perspective, the knowledge gained through this study may further direct how stroke survivors should be trained early onwards. This may eventually result in faster reaching of independence in daily life activities to enable patients to engage as early as possible in more intensive, semi-supervised therapies (72) and supported discharge (73).

However, implications may go far beyond clinical rehabilitation practice alone. An improved understanding of recovery that distinguishes behavioral restitution from compensation will contribute to the design of rehabilitation devices as well as development of sensitive measurements of quality of movement. The latter will improve future trial design regarding the choice of outcome measures (16). Specifically, addressing effectiveness of novel behavioral and pharmacological treatments based on such measures is warranted (35). Moreover, interpretation of neuroimaging may greatly benefit from this knowledge. Current literature argues the importance of knowledge about the associations between behavioral improvements and changes in brain activity and connectivity (35), yet the neural correlates of behavioral restitution remain so far unknown.

### Study Limitations

The study described in the current report also has some limitations. Although the number of participants that will be included in the current study is greater as compared to previous prospective balance recovery studies (30, 34, 40, 42, 68) [with ranges between *n* = 13 (68) and *n* = 37 (40)], the desired sample size is limited. Second, since we use one specific balance condition it is not possible to determine whether results can be generalized to other balance tasks. Recent literature suggests that increasing challenges may reduce the degree of asymmetry in bipedal balance control (74) and balancing in everyday life environments requires rather reactive control skills (11). The results may therefore not fully capture the upper boundary of neurological recovery at the hemiplegic side and translation to dynamic balance conditions remains unknown. However, incorporating more-challenging paradigms may lead to a greater amount of missing values since many subacute patients with hemiparesis are not able to safely withstand perturbations when standing (34, 42) or perform dynamic tasks such as walking (75) until several weeks after stroke. Third, repeated measurements were performed with either force-plates or a mobile pressure-plate system. This enables us to perform measures in various (clinical) settings and recruit more broadly. However, while both systems can extract bilateral COP profiles, technical variations differ. To control for this, focus lies on within-subjects time series analyses and, additionally, we will add “equipment” as a covariate to our regression models. Fourth, measurements are restricted to the first 12 weeks. Yet, it might be of interest to further continue measurements acknowledging that few studies revealed that about 15% of survivors improve (76) and 25% will deteriorate beyond the first 6 months (77). Lastly, the study did not monitor the type and amount of therapy provided to each participant and we are unable to correct for these factors. The lack of using uniform guidelines for stroke rehabilitation in Flanders, Belgium could lead to differences in how subjects are treated in cooperating facilities. Moreover, mildly-affected subjects may be discharged earlier and receive less intensive outpatient therapy afterwards. However, evidence that current rehabilitation interventions impact neurological recovery is still lacking so far (17, 78), particularly if provided as part of standard care which is shown to be low-dosed (79, 80). Since usual care has not been systematically modified, differences in rehabilitation treatment are expected to have a limited impact on outcomes of the current study.

## Author Contributions

JS, ST, and WS participated in the planning and initial conception of this study. GK and LY participated in further conceptual work and designing the study. JS wrote the initial manuscript, is responsible for the study process and day-to-day study management under guidance of WS. All authors were involved in drafting the manuscript and have approved the final version for publication.

## Funding

SJ receives funding by the Fonds Wetenschappelijk Onderzoek (FWO), Flanders, Belgium in order to execute the described research project and communicate results (application nr.: 1S64819N).

## Conflict of Interest

The authors declare that the research was conducted in the absence of any commercial or financial relationships that could be construed as a potential conflict of interest.

## Publisher's Note

All claims expressed in this article are solely those of the authors and do not necessarily represent those of their affiliated organizations, or those of the publisher, the editors and the reviewers. Any product that may be evaluated in this article, or claim that may be made by its manufacturer, is not guaranteed or endorsed by the publisher.
